# Supplementary material for: Effects of Nitrogen Supply on Induced Defense in Maize (Zea mays) against Fall Armyworm (Spodoptera frugiperda)
Source: Int J Mol Sci. 2022 Sep 9;23(18):10457. doi: 10.3390/ijms231810457 (PMC9504019; doi:10.3390/ijms231810457)
Supplement: Supplementary file 1 [file ijms-23-10457-s001.zip › ijms-1891862-supplementary.pdf]

Supplementary Materials

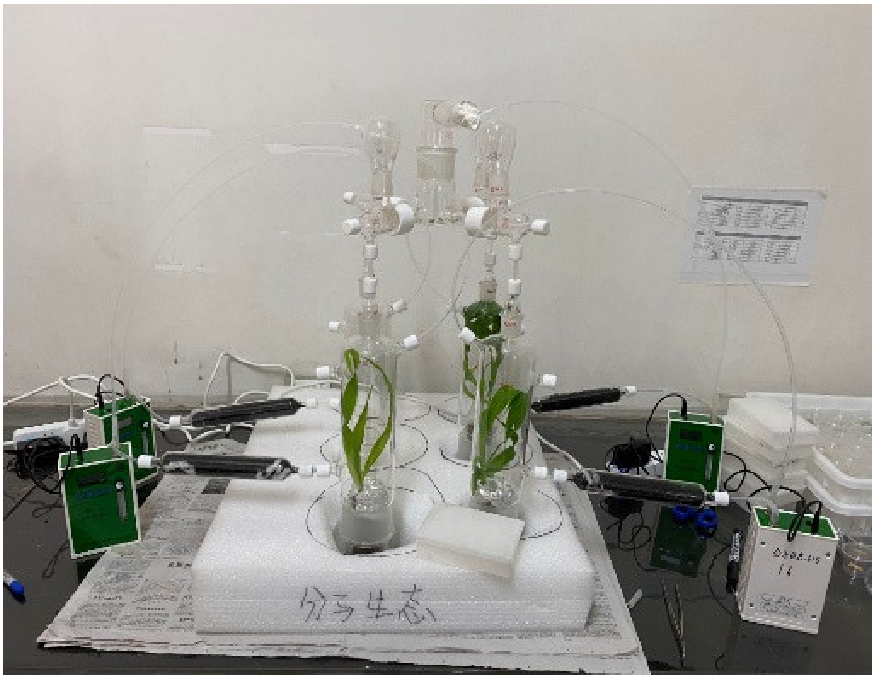

Figure S1. Olfactometer choice of *S. frugiperda* larvae in four-arm olfactometer.

Table S1. The olfactometer bioassays conducted in this study

Bioassay	Arm 1	Arm 2	Arm 3	Arm 4
1	N0H	N1H	N2H	air
2	N1SF	N1SF	N1SF	air
3	N2SF	N2SF	N2SF	air
4	N0H	N0SF	N0JA	air
5	N1H	N1SF	N1JA	air
6	N2H	N2SF	N2JA	air
7	N0H	N1H	N2H	air
8	N0SF	N1SF	N2SF	air
9	N0JA	N1JA	N2JA	air

Table S2. Effects of N supply on the composition and relative abundance of volatiles emitted by maize plants from different induced treatments

No.	Peak RT (min)	Chemical name	Relative abundance of volatiles in maize plants (%)								
			N0H	N1H	N2H	N0SF	N1SF	N2SF	N0JA	N1JA	N2JA
1	19.065	Caprolactam	0.349±0.0	−	0.12±0.0	1.19±0.0	−	−	0.1±0.0	−	−
2	18.173	Cyclohexylamine	−	−	0.096±0.0	0.09±0.0	−	0.12±0.0	−	0.089±0.007	0.06±0.0
3	23.68	Cyclopropylamine	−	−	−	−	0.254±0.0	−	−	−	−
4	32.1	Propionamide	−	−	0.066±0.0	−	−	−	−	−	−
5	25.309	Phenol	0.396±0.066a	0.213±0.035a	0.222±0.059a	0.34±0.104a	0.322±0.044a	0.287±0.019a	0.18±0.028b	0.196±0.019ab	0.237±0.007a
6	20.325	Indoles	0.371±0.069*	0.26±0.0	−	−	−	−	−	0.412±0.019	2.185±1.44*
7	5.51	Paraxylene	−	0.87±0.0*	0.13±0.075	0.14±0.0b	0.43±0.106ab	0.645±0.201a	0.05±0.0b	0.69±0.205a	0.20±0.075ab
8	26.315	Dibutyl hydroxy toluene	−	0.695±0.102	1.068±0.0*	−	−	−	−	0.701±0.0	0.55±0.0
9	31.17	Biphenyl	−	−	0.07±0.0	−	−	−	−	−	−
10	16.55	Naphthalene	−	−	−	0.18±0.0	−	0.34±0.0	0.04±0.0	−	0.29±0.0*
11	32.545	Hydroquinone	−	0.13±0.014	0.206±0.0	−	0.123±0.019	0.12±0.0	0.38±0.0a	0.138±0.001b	0.17±0.0b
12	26.67	Pyrocatechol	−	−	−	−	0.095±0.0	0.22±0.0*	0.16±0.0	0.1±0.0	−
13	3.915	Hexanone	0.323±0.014a	0.157±0.057b	0.152±0.045b	0.39±0.091a	0.358±0.044a	0.297±0.027a	0.105±0.031a	0.198±0.629a	0.187±0.053a
14	3.865	3-hexanone	0.365±0.059a	0.197±0.062b	0.175±0.05b	0.373±0.061a	0.427±0.065a	0.377±0.020a	0.14±0.033b	0.234±0.069a	0.22±0.121a
15	3.928	2-Heptanone	−	0.27±0.113	0.119±0.072		0.458±0.086	0.553±0.022	0.15±0.0a	0.16±0.0a	0.21±0.014a
16	6.035	Cyclohexanone	−	0.07±0.0	0.395±0.064*	0.38±0.219		0.943±0.332*	0.13±0.042b	0.141±0.0b	0.655±0.672a
17	13.978	Cyclohexenone	−	0.295±0.109	1.41±0.905*	2.95±0.0a	1.028±0.545b	0.545±0.159c	1.92±0.0a	0.56±0.159b	0.26±0.0b
18	8.527	6-Methyl-5-hepten-2-one	0.38±0.0	−	−	−	0.3±0.0	0.303±0.045	−	−	−
19	27.022	2-Coumaronone	−	−	−	0.91±0.431a	0.782±0.194a	0.635±0.131a	−	−	0.31±0.0
20	35.359	2-Pentadecanone	2.2±0.0*	0.063±0.11	−	0.65±0.0	−	−	−	0.402±0.0	−

21	28.575	Cycloheptanone	−	0.027±0.046	−		−	−	−	−	−
22	35.475	Pentadecanone	−	0.1±0.173	0.16±0.138	3.25±0.0a	1.132±0.333b	0.685±0.074c	−	0.53±0.042	0.365±0.134
23	5.315	2-Butanone	−	−	0.017±0.029	−	−	−	−	−	−
24	28.532	Cyclopentanone	−	−	−	−	−	−	−	−	0.07±0.0
25	25.644	Methyl vinyl ketone	−	−	−	−	−	0.12±0.0	−	0.08±0.0	−
26	13.374/7.835[a]	Benzaldehyde	0.67±0.0*	−	0.139±0.05	0.13±0.0a	0.19±0.0a	0.215±0.053a	0.08±0.0b	0.2±0.045a	0.07±0.0b
27	10.955	Phenylacetaldehyde	−	−	−	0.16±0.0a	0.15±0.0a	0.21±0.035a	0.08±0.0	0.088±0.004	−
28	13.463	Nonanal	0.83±0.0a	0.32±0.147b	0.498±0.122b	0.3±0.0b	0.61±0.885a	0.657±0.138a	0.21±0.0b	0.381±0.109b	0.555±0.258a
29	20.665	Tridecanal	−	−	−	−	−	0.1±0.0	0.09±0.0	−	−
30	9.221	Caprylic aldehyde	−	−	0.19±0.0	0.08±0.0	−	0.22±0.0*	−	0.07±0.0	−
31	17.277	Decanal	0.45±0.0a	0.163±0.048b	0.34±0.028a	0.335±0.053a	0.325±0.010a	0.4±0.079a	−	0.32±0.063	0.35±0.183
32	21.735	Myristic aldehyde	−	0.017±0.029	0.038±0.066	−	0.093±0.0	0.16±0.049*	−	0.083±0.0	0.12±0.0
33	19.13	Decenal	−	−	0.088±0.102	−	0.115±0.0	0.13±0.0	−	−	0.09±0.0
34	39.69	1-Heneicosanol	0.304±0.0	−	−	−	−	−	−	−	−
35	32.661	2-Isopropyl-5-methyl-1-Heptanol	0.318±0.0	−	−	0.11±0.0	−	−	−	−	−
36	5.625	1-Butanol	−	−	0.22±0.381	−	0.089±0.0	0.38±0.0*	0.06±0.0c	0.129±0.0b	0.48±0.0a
37	7.215	2-Pentanol	−	0.077±0.133	−	−	−	−	0.04±0.0b	0.09±0.0b	0.14±0.0a
38	10.6/26.31[a]	Benzyl alcohol	1.781±0.79a	0.06±0.104c	0.429±0.575b	4.155±0.0a	3.76±1.470a	0.85±0.601b	0.925±0.017	0.819±0.092	−
39	12.17/21.916[a]	1-Octanol	−	0.54±0.0	0.43±0.141	−	0.955±0.378	0.67±0.0	0.055±0.017	0.465±0.234*	−
40	12.51	1-Nonanol	−	−	−	−	−	−	0.03±0.0	−	−
41	16.795/23/25.53[a]	Dodecanol	1.47±0.0	−	−	−	0.091±0.0	0.89±0.0	0.08±0.0212	−	−
42	10.36/30.48[a]	Hexyl alcohol	0.84±0.0a	0.69±0.348a	0.194±0.0b	0.55±0.311b	1.575±0.851a	0.67±0.0b	0.077±0.133	−	−
43	10.131	n-Propyl Alcohol	0.69±0.0*	0.08±0.0	−	0.31±0.127a	0.514±0.104a	0.407±0.193a	−	−	−
44	10.185	propan-2-ol	−	0.17±0.071	−	0.28±0.240a	0.24±0.078a	0.24±0.049a	−	−	−
45	10.345	2-Propyl-1-pentanol	−	−	0.288±0.0	4.63±0.0a	1.218±0.0b	4.31±0.0a	−	−	2.61±0.0

46	27.591	1,2-hexadecanediol	−	−	−	0.31±0.0			−	−	−
47	23.9	Cedrol	−	0.07±0.0	0.112±0.0*	−	0.156±0.0	0.29±0.022*	−	0.091±0.007	0.24±0.0*
48	6.206	2-Heptanol	−	−	−	−	0.207±0.0	0.255±0.010	−	−	−
49	12.395	2-butanol	−	0.47±0.0	−	−	0.089±0.0	−	−	−	−
50	24.39	Bisabolol	−	−	−	−	0.119±0.0	−	−	−	−
51	39.315	Nerolidol	−	−	−	−	0.116±0.0	−	−	−	0.06±0.0
52	37.194	Cyclohexanol	−	−	−	−		−	−	−	0.19±0.0
53	35.266	Oleyl alcoho	−	−	0.0915±0.026	−	1.2±0.0*	0.47±0.134	−	0.35±0.120	0.21±0.0
54	33.53	Tridecyl alcohol	−	−		−			−		0.09±0.0
55	35.719	Phthalic acid	0.99±0.55a	0.767±0.262a	0.897±0.341a	1.29±0.572a	0.993±0.183a	1.305±0.258a	0.11±0.035b	0.601±0.143a	0.823±0.420a
56	16.36	Caprylic Acid	−		0.102±0.0				0.06±0.0	0.151±0.007*	
57	15.118	Acetic Acid	−	0.145±0.120	0.48±0.0*	0.27±0.0		0.35±0.070	0.35±0.0b	0.296±0.071b	0.765±0.456a
58	18.007	Acrylic acid	−	0.28±0.08	1.563±1.241*	5.25±0.0a	1.231±0.650b	1.377±0.811b	−	0.164±0.022	1.185±0.682*
59	18.461	Adipic acid	−	−	−	0.07±0.0	−	−	−	−	−
60	25.107	Methylnonanoic acid	−	−	−	0.78±0.0	−	−	−	−	−
61	22.62	Behenic acid	−	−	0.334±0.159	0.021±0.0	−	0.74±0.0*	−	0.099±0.0	0.3±0.0*
62	28.43	Propanoic acid	−	0.08±0.0	0.156±0.0*	0.42±0.0a	0.135±0.0b	0.35±0.0a	−	−	0.18±0.0
63	18.936/37.37[a]	Sulfurous acid	−	0.11±0.0	0.211±0.0	0.2±0.0*	0.103±0.0	−	−	0.07±0.0	0.08±0.0
64	36.961	Palmitic acid	0.72±0.0	0.89±0.127	−	0.727±0.245a	1.73±0.827a	0.49±0.0a	2.29±0.0a	0.99±0.230b	1.57±0.035a
65	37.679	Pentadecanoic acid	−	0.06±0.0	−	1.2±0.0	1.11±0.0	−	−	0.982±0.0	−
66	38.386	Metaupon	−		−	0.13±0.0	−	−	−	−	−
67	27.265	Pentadecenoic acid	−	0.08±0.0	−	−	−	−	−	−	−
68	22.62	Dodecanedioic Acid	−	0.11±0.0	−	−	0.689±0.056	−	−	0.09±0.0	−
69	37.06	3-Phenylpropanoic acid	−	−	0.109±0.0	−	0.101±0.0	−	−	−	−
70	27.888	Lauric acid	−	0.05±0.0	0.189±0.088*	0.3±0.0	−	−	−	0.26±0.0	−
71	27.34	Heptadecenoic acid	−	−	−	−	−	−	−	−	0.41±0.0

72	32.173	Cyclopentaneacetic acid	–	–	–	–	–	0.35±0.0	–	–	0.41±0.0
73	18.405	Adipic acid	–	–	–	–	–	0.11±0.0	–	–	–
74	19.515	Sulphurous acid	–	–	0.211±0.0	–	–	–	–	–	–
75	24.085	β-Caryophyllene	–	–	–	–	0.2±0.0	–	0.13±0.0	–	–
76	13.838	Heptadiene	–	–	–	0.24±0.0	0.39±0.014	–	–	0.16±0.0	–
77	23.981	Cedrene	–	–	–	0.15±0.0	–	0.18±0.0	–	–	–
78	37.366	1-hexadecene	–	–	–	0.08±0.0	–	–	–	–	–
79	10.375	Limonene	–	–	–	0.36±0.0a	0.315±0.0a	0.47±0.0a	–	4.27±1.156*	0.135±0.025
80	8.736	β-Laurene	–	–	–		0.132±0.0	0.33±0.014	–	0.19±0.0	0.33±0.014*
81	13.355	1,6-Octadiene	–	–	0.27±0.0	0.6±0.0	–	–	–	–	–
82	8.735	Geranyl bromide	–	0.08±0.0	0.27±0.0*	–	–	–	0.04±0.0	–	0.89±0.0*
83	34.961	Isopropyl myristate	–	0.67±0.0	–	–	0.815±0.427	1.145±0.300	0.06±0.0	–	–
84	34.97/29.76[a]	Vinyl myristate	–	–	–	0.23±0.0	–	–	–	–	–
85	38.605	Isopropyl palmitate	–	0.36±0.0	–	–	0.099±0.0	–	–	–	–
86	18.065	Methyl Heptafluorobutyrate	–	–	–	–	–	–	–	–	0.1±0.0
87	15.806	Methyl salicylate	–	–	–	–	–	0.19±0.0	–	–	–
88	18.92	γ-caprolactone	–	–	–	–	–	0.09±0.0	–	–	–
89	32.174	Jasmonates	–	–	–	–	–	0.41±0.0	–	–	0.71±0.0
90	19.61	Dodecane	0.27±0.0b	0.31±0.0b	0.51±0.0a	–	–	–	–	–	–

"-" in the tables means undetected.

[a] means that volatile was found at multiple time points.

Values followed by the same lowercase letters in the same row were not significantly different among three N concentrations under the same induced treatments (ANOVA). Values followed by asterisk "*" indicates significant differences between two N concentrations under the same induced treatments (T-test).
